# Peripheral blood CD3+HLADR+ cells and associated gut microbiome species predict response and overall survival to immune checkpoint blockade

**DOI:** 10.3389/fimmu.2023.1206953

**Published:** 2023-08-29

**Authors:** Joao Gorgulho, Christoph Roderburg, Fabian Beier, Carsten Bokemeyer, Tim H. Brümmendorf, Tom Luedde, Sven H. Loosen

**Affiliations:** ^1^ Department of Oncology, Hematology and Bone Marrow Transplantation with Section of Pneumology, University Medical Centre Hamburg-Eppendorf, Hamburg, Germany; ^2^ Mildred Scheel Cancer Career Center, University Cancer Center Hamburg, University Medical Center Hamburg-Eppendorf, Hamburg, Germany; ^3^ Department of Gastroenterology, Hepatology and Infectious Diseases, University Hospital Düsseldorf, Medical Faculty of Heinrich Heine University Düsseldorf, Düsseldorf, Germany; ^4^ Center for Integrated Oncology Aachen-Bonn-Cologne-Düsseldorf (CIOABCD), Aachen, Germany; ^5^ Department of Medicine IV, University Hospital Rheinisch Westfällisch Technische Hochschule (RWTH) Aachen, Aachen, Germany

**Keywords:** PD-1, HLA-DR, checkpoint inhibitors, microbiome, prognosis, biomarker

## Abstract

**Background:**

The search for biomarkers to identify ideal candidates for immune checkpoint inhibitor (ICI) therapy is fundamental. In this study, we analyze peripheral blood CD3+HLADR+ cells (activated T-cells) as a novel biomarker for ICI therapy and how its association to certain gut microbiome species can indicate individual treatment outcomes.

**Methods:**

Flow cytometry analysis of peripheral mononuclear blood cells (PBMCs) was performed on n=70 patients undergoing ICI therapy for solid malignancies to quantify HLA-DR on circulating CD3+ cells. 16s-rRNA sequencing of stool samples was performed on n=37 patients to assess relative abundance of gut microbiota.

**Results:**

Patients with a higher frequency of CD3+HLADR+ cells before treatment initiation showed a significantly reduced tumor response and overall survival (OS), a worst response and experienced less toxicities to ICI therapy. As such, patients with a frequency of CD3+HLADR+ cells above an ideal cut-off value of 18.55% had a median OS of only 132 days compared to 569 days for patients below. Patients with increasing CD3+HLADR+ cell counts during therapy had a significantly improved OS. An immune signature score comprising CD3+HLADR+ cells and the neutrophil-lymphocyte ratio (NLR) was highly significant for predicting OS before and during therapy. When allied to the relative abundance of microbiota from the Burkholderiales order and the species Bacteroides vulgatus, two immune-microbial scores revealed a promising predictive and prognostic power.

**Conclusion:**

We identify the frequencies and dynamics of CD3+HLADR+ cells as an easily accessible prognostic marker to predict outcome to ICIs, and how these could be associated with immune modulating microbiome species. Two unprecedented immune-microbial scores comprising CD3+HLADR+, NLR and relative abundance of gut bacteria from the Burkhorderiales order or Bacteroides vulgatus species could accurately predict OS to immune checkpoint blockade.

## Introduction

Immune checkpoint inhibitors (ICI) contributed to a drastic change in the landscape of cancer therapy, giving hope to many advanced cancer patients, which are now able to achieve improved response and overall survival (1–3). Currently, more than 8 different such agents have been approved for a wide spectrum of cancer entities (4). Nonetheless, many patients only experience toxicities and/or fail to respond to ICI therapy. The question, which patients would mostly benefit from immune checkpoint blockade, remains yet unanswered, despite countless studies identifying different biomarker candidates. Among these are peripheral blood-based biomarkers such as specific lymphocyte subpopulations (5) and the neutrophil-to-lymphocyte ratio (NLR) (6), as well as the relative abundance of diverse taxa in the gut microbiome with a certain heterogeneity across cohorts (7).

The human leukocyte antigen-DR isotype (HLA-DR) is a major-histocompatibility complex class II (MHC-II) molecule present on the surface of antigen presenting cells (APCs), which together with a foreign peptide constitute a ligand for T-cells and engage T-cell response. It is known as an immune stimulation and late activation marker (8) for T-cells. CD3+HLADR+ cells are deemed as activated T lymphocytes, which are upregulated in autoimmune diseases (9) and HIV infection (10). In cancer patients they have had divergent results, with high CD3+HLADR+ levels being associated with shorter relapse-free survival in Hodgkin and non-Hodgkin lymphoma (11, 12), but with better response to neoadjuvant therapy in breast cancer (13). Studies assessing HLA-DR expression on lymphocytes as a potential biomarker for ICI therapy are scarce and focus mainly on single tumor entities (14).

The gut microbiome, which shows tremendous immune modulatory effects, mediated through different species (7), has recently emerged as another field of interest in terms of predicting response to immune checkpoint blockade. An active manipulation of the human microbiome through dietary interventions (15) or fecal microbiota transplantation (FMT) seems to increase efficacy to ICI therapy, and can even, in some cases, overcome a prior resistance to PD-1 and CTLA-4 antibodies (16). However, an association between activated T cells and specific microbiome species have, to our knowledge, not been studied. In the present analysis, we evaluate the prognostic role of CD3+HLADR+ cell frequencies and its dynamics during ICI therapy and analyze how they correlate with the relative abundance of microbiome species that could influence HLA-DR expression.

## Patients and methods

### Study population

70 patients with advanced stage solid neoplasia were prospectively recruited at the interdisciplinary cancer outpatient clinic at the University Hospital RWTH Aachen from August 2017 to September 2019 (see **Table 1** for patient characteristics) before undergoing ICI therapy, as described before (17). The study was conducted in accordance with the ethical standards laid down in the 1964 Declaration of Helsinki and its later amendments and the protocol was approved by the ethics committee of the University Hospital RWTH Aachen, Germany (EK 206/09) with all patients delivering written informed consent.

**Table 1:** Patient characteristics.

Parameter	Study cohort	Subgroup of patients for microbiome analysis
**Cancer patients**	n=70	n=37
Sex [%]:		
male-female	70.0 - 30.0	64.9 – 35.1
Age [years, median and range]	67.0 [38-87]	67.4 [38-87]
BMI [kg/m^2^, median and range]	24.4 [15.9-42.3]	25.2 [15.9-40.0]
Tumor entity [%]		
NSCLC Melanoma Urogenital tract GIT Head and neck Other malignancies	34.2 20.0 12.9 14.3 10.0 8.6	29.7 29.7 13.5 10.8 5.4 10.8
Staging [%]		
UICC III UICC IV	10.0 90.0	13.5 86.5
ECOG PS [%]		
ECOG 0 ECOG 1 ECOG 2 ECOG 3	7.1 54.2 37.2 1.5	13.5 59.5 27.0 0.0
Therapeutic agent [%]		
Nivolumab monotherapy Pembrolizumab monotherapy Nivolumab/Ipilimumab Other (Avelumab, Durvalumab)	61.4 22.9 8.6 7.1	59.5 16.2 13.5 10.8
Smoker status [%]		
Never Yes, ex Yes, present Unknown	10.0 41.4 14.3 34.3	13.5 37.8 10.8 37.8
Prior therapy [%]		
Yes No	67.1 32.9	56.9 43.2
Side effects [%]		
Any CTC G3 or higher	38.6 7.1	45.9 10.8

BMI, body mass index; ECOG PS, “Eastern Cooperative Oncology Group” performance status, NSCLC, non-small cell lung cancer; GIT, gastrointestinal tract; CTC, common toxicity criteria.

### Determination of response to ICI therapy

Patients were regularly consulted by a trained oncologist prior to each therapy cycle. Determination of response to ICI therapy was based on clinical and radiological evaluation by CT scan approximately every three months, evaluated by at least two independent experienced radiologists. Based on the assessment, patients were stratified into two groups: patients with a complete response (CR), partial response (PR) and stable disease (SD) were included in the “disease control” (DC) group, while the ones who exhibited progressive disease (PD) were enrolled in the “non-DC” group.

### Assessment of peripheral PBMC subsets

One peripheral blood EDTA tube was drawn per patient (n=70) prior to ICI therapy initiation, at an early (after one to two cycles, n=51) and late time-point (after three to five cycles, n=47) during therapy. Freshly isolated cells were lysed using the Immunoprep Reagent System (Beckman Coulter) and staining was performed with two different flow cytometry panels. Panel 1 was stained with the antibody mix CD45-FITC/CD56-PE/CD19-ECD/CD3-PC5, to which the antibody CD-16 PE was added, and panel 2 was stained with the antibody mix CD45-FITC/CD4-PE/CD8-ECD/CD3-PC5, to which the antibody HLA-DR-PC7 was added (all antibodies from Beckman Coulter, Krefeld, Germany), according to manufacturer´s instructions. Flow-cytometry analysis was carried out and analyzed using NAVIOS cytometer and analysis software (Beckman Coulter). These analyses were performed within the clinical routine diagnostics of immune status by the hematological laboratory of the department of medicine IV of the University Medical Center Aachen, which includes standardized gating strategy to distinguish B cells (CD19+), NK cells (CD3-CD56+CD16+), and T cell subsets (CD3+CD4+, CD3+CD8+, CD3+CD56+CD16+, CD3+HLA-DR+) (**Supplementary Figures 1A–D**).

### 16s rRNA sequencing of stool samples and amplicon sequence analysis

Stool samples were obtained from n=37 patients before initiation of therapy and from n=15 patients after three to five cycles during therapy using a stool collection tube with 8ml DNA stabilization Buffer (Stratec Molecular GmbH, Berlin, Germany) and frozen aliquots were preserved at -80°C until further processing. Samples were sequenced at the ZIEL Institute for Food & Health Core Facility Mikrobiom/NGS (Freising, Germany) according to methods described before (18). Shortly, bead-beating and heat-treatment were used for cell lysis and gDNA columns (Macherey-Nagel, Düren, Germany) were employed to purify metagenomic DNA. The V3/V4 region of 16 S ribosomal RNA (rRNA) genes was amplified (25 cycles) from 24 ng DNA using primers 341F and 785R49. After purification using the AMPure XP system (Beckmann Coulter Biomedical GmbH), sequencing was carried out in paired-end mode (PE275) with pooled samples using a MiSeq system (Illumina, Inc., San Diego, California, USA) following the manufacturer’s instructions and a final DNA concentration of 10 pM and 15% (v/v) PhiX standard library. The generated raw read files were pre-processed using the IMNGS platform (19), a pipeline based on the UPARSE approach (20) to build sample-specific sequence databases and OTU-based profiles. We then further analyzed generated data using the Rhea pipeline in R studio version 1.2.5, a set of R scripts for analysis of Operational Taxonomic Units (OTUs) (21). Only OTUs with a relative abundance > 0.5% total sequences in at least one sample were further analyzed. For precise identification of certain OTU sequences, the EzBioCloud database was used (22).

### Statistical analysis

Shapiro-Wilk-Test was used to check for normal distribution of the data. By employing the Mann-Whitney-U-Test and Kruskal-Wallis-H-Tests, non-parametric data were compared. The median, quartiles and ranges of these data are displayed in box plot graphics. Kaplan-Meier curves aided in demonstrating the influence of a specific parameter on OS. The Log-rank test was used to evaluate statistical differences between subgroups. Repeated measures ANOVA was used for longitudinal analyses of CD3+HLADR+ frequencies at the three time-points (before ICI treatment, early and late time-point), reporting the main F-test. For calculation of the optimal cut-off of CD3+HLADR+ cell frequencies and counts, NLR and relative abundances of specific microbiome taxa to discriminate between short- and long-term survivors, the “Charité cut-off finder” was applied, which fits Cox proportional hazard models to the dichotomized survival status (deceased or alive) as well as the survival time (duration between first ICI administration and death/last follow-up) and defines the optimal cut-off as the value with the most significant split in log-rank test (23). In addition, uni- and multivariate Cox-regression was performed with parameters with a p-value of <0.100 in univariate testing being included into multivariate testing. The hazard ratio (HR) and the 95% confidence interval are displayed. Gut microbiome analysis was performed using the Rhea pipeline (21), mainly the normalization (to account for differences in sequence depth), beta- (computed based on generalized UniFrac distances) (24) and alpha-diversity (on the basis of species richness and Shannon effective diversity) (25), as well as taxonomic binning steps (using SILVA and RDP classifier) (26, 27). The Spearman correlation coefficient was used for correlation analyses between flow cytometry data and relative abundances of specific taxa in the gut microbiome. All statistical analyses were performed using SPSS 23 and 25 (SPSS, Chicago, IL, USA) and R studio version 1.2.5 (Posit PBC, Boston, MA, USA). A p-value of < 0.05 was considered statistically significant (* p < 0.05; ** p < 0.01; *** p < 0.001).

## Results

### Characteristics of the study population

70 patients with advanced solid malignancies receiving ICI therapy were included (detailed characteristics are shown in **Table 1**). The median age was 67.0 years (range 38 to 87 years).; 70.0% were males. The predominant cancer entity was NSCLC (34.2%), followed by malignant melanoma (20.0%), urogenital cancer (12.9%), GI-cancers (14.3%), head and neck tumors (10.0%) and others (8.6%). Only patients in UICC stadium III (10.0%) and IV (90.0%) were recruited. Immune related adverse effects (IRAE) of any grade were experienced by 38.6% of patients and 7.1% experienced IRAE graded ≥3. All patients were treated with immune checkpoint inhibitors only. Of all 70 patients, a subgroup of 37 and 16 patients were available for stool microbiome analyses at baseline and at 3 months after treatment initiation (see **Table 1**).

### Baseline frequencies of peripheral blood CD3+HLA-DR+ cells significantly predict toxicity, response at 6 months and survival at 6 months after initiation of immune checkpoint blockade

**Figure 1:**
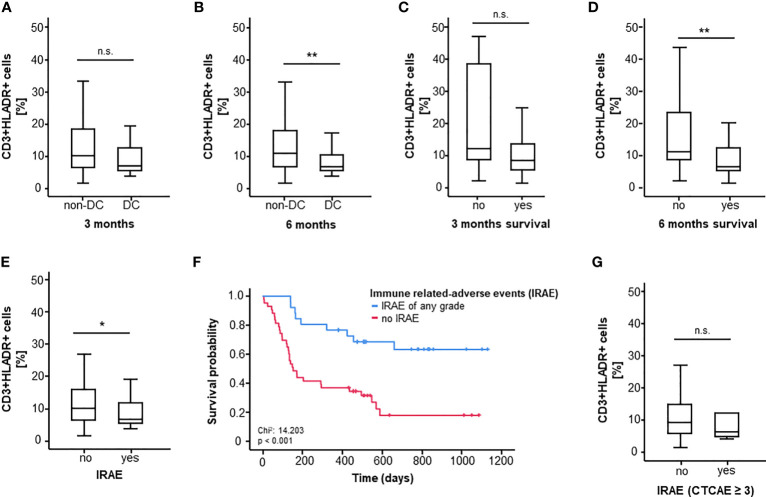
CD3+HLADR+ cell frequencies before ICI therapy significantly predict OS, response and toxicity. **(A, B)** High CD3+HLADR+ cell frequencies in the peripheral blood at baseline indicate a worse response to ICI therapy at 3 and 6 month (p_3months_=0.051, p_6months_=0.008). **(C, D)** High baseline CD3+HLADR+ cell frequencies indicate poor 3 and 6 months survival under immune checkpoint blockade (p_3months_=0.067, p_6months_=0.003). **(E)** Baseline CD3+HLADR+ cell frequencies are higher among patients who develop immune related adverse events (IRAE) under ICI therapy (p=0.043). **(F)** Overall survival is lower in patients who do not develop any grade of IRAE (median OS: 151 days vs. “not reached”, p<0.001). **(G)** Baseline frequencies of CD3+HLADR+ cells do not differ between patients experiencing IRAE ≥ grade 3 and patients who do not (p=0.595). *: significant (p<0.05); **: highly significant (p<0.01); n.s.: not significant (p>0.05).

First, we assessed differences between pretreatment CD3+HLADR+ cell frequency between ICI-responders (DC) and non-responders (non-DC) three and six months after therapy initiation. In this case, only a non-significant trend towards a higher CD3+HLADR+ cell frequency in non-responders compared to responders could be observed after 3 months, however a clear association between a better therapy response and lower pretherapeutic CD3+HLA-DR+ cell frequencies (p_3months_=0.051, p_6months_=0.008, **Figures 1A, B**) was observed. When looking at 3- and 6-months-survival, patients who were still alive six months after treatment initiation had a significantly lower initial CD3+HLA-DR+ cell frequency compared to non-survivors (p=0.003, median_survivor_=6.65%, median_deceased_=11.15%), while a similar trend towards lower CD3+HLADR+ cell frequency among patients who died within the first three months became apparent (p=0.067, **Figure 1C, D**).

In a further step, we looked at differences in CD3+HLADR+ cell frequencies with respect to treatment-related adverse events. Interestingly, patients not experiencing IRAE of any grade had significantly higher CD3+HLADR+ cell frequencies (median_yes_=6.60% vs. median_no_=10.00%, p=0.043, **Figure 1E**). Notably, in our cohort, patients who experienced IRAE of any type showed an improved overall survival (p<0.001, **Figure 1F**). However, the presence of HLA-DR expressing T cells before treatment failed to predict iRAE graded 3 or higher among all patients (p=0.595, **Figure 1G**).

### Frequencies of peripheral blood CD3+HLADR+ cells are comparable across different clinical characteristics but associated with the ECOG performance status and can be influenced by the ICI regimen

For further characterization of the predictive role of CD3+HLADR+ values in ICI therapy, these were evaluated according to different clinical characteristics. Regarding tumor entity, sex, tumor stadium (UICC), smoking status and whether patients had previous lines of systemic therapy, no significant differences could be observed (**Supplementary Figures 2A–E**). Notably, there was a significantly higher peripheral blood CD3+HLADR+ frequency in patients with a higher ECOG performance status compared to patients with a lower ECOG performance status (p=0.026, **Supplementary Figure 2F**). Despite observing no significant difference between CD3+HLADR+ cell frequencies at baseline with respect to the administered ICI agent (**Supplementary Figure 2G**), HLADR+ cell frequencies were significantly higher at the early time-point in patients receiving a combined anti-PD-1/CTLA immune checkpoint blockade with Nivolumab and Ipilimumab (p=0.036, **Supplementary Figure 2H**).

### Pretreatment circulating CD3+HLADR+ frequencies are an independent predictor of overall survival to ICI therapy

**Figure 2:**
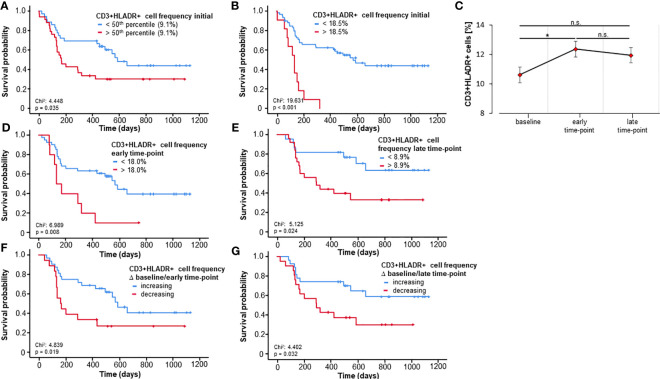
Baseline and longitudinal frequencies of circulating CD3+HLADR+ cells predict overall survival to immune checkpoint blockade. **(A)** Patients with baseline CD3+HLADR+ cell frequencies above the median (9.1%) have a significantly impaired overall survival (OS, p=0.035). **(B)** A baseline CD3+HLADR+ cell frequency above the ideal cut-off value (18.5%) indicate a significantly reduced median OS (132 vs. 569 days, p<0.001). **(C)** Frequencies of CD3+HLADR+ cells significantly increase from baseline to the early-time point (p=0.015) but remain unaltered thereafter (error bars indicate SEM). **(D, E)** Patients with CD3+HLADR+ cell frequencies above the ideal cut-off at the early and late time-point have a significantly impaired OS (p_early_=0.008, p_late_=0.024). **(F, G)** Increasing CD3+HLADR+ cell frequencies between baseline and the early or late time-point indicate a better outcome (p_baseline/early_=0.019, p_baseline/late_=0.032). *: significant (p<0.05); **: highly significant (p<0.01); n.s.: not significant (p>0.05).

Based on the predictive power of activated T cells regarding 3- and 6-months-survival, we next took a deeper look at the prognostic role of these cells with respect to OS using Kaplan-Meier-curve estimates. In a first step, patients were split into two groups based on the median frequency of these cells. Interestingly, the median CD3+HLADR+ frequency (9.1%) at baseline significantly discriminated between short- and long-term survivors (p=0.035, **Figure 2A**). Since the median is likely not ideal to discriminate patients regarding OS, we then applied the Charité cut-off finder (further described in Patients and Methods) to establish a prognostically highly relevant cut-off value for CD3+HLADR+ cell frequencies (18.5%). Patients with a pretreatment CD3+HLADR cell frequency below this ideal cut-off survived significantly longer (median 569 days) than patients above this threshold (median 132 days, p<0.001, **Figure 2B**).

For further characterization of the role of CD3+HLADR+ cell frequencies as independent predictors of OS, we applied uni- and multivariate Cox-regression analyses. Univariate Cox-regression further sustained our hypothesis that the frequency of CD3+HLADR+ cells acts as a potent prognostic predictor in patients undergoing immune checkpoint blockade (HR: 1.068 [95%CI: 1.037 – 1.099], p<0.001, **Table 2**). Next, we included several prognostically relevant parameters such as CD3+CD8+ cell frequencies at baseline, ECOG PS, Hemoglobin, AST and ALT (p<0.110, **Table 2**) into multivariate Cox-regression analysis, which revealed peripheral blood CD3+HLADR+ cell frequencies as an independent predictor for OS in patients before commencement of ICI therapy (HR: 1.054 [95%CI: 1.007-1.103], p=0.024, **Table 2**).

**Table 2:** Uni- and multivariate Cox-regression analysis for the prediction of overall survival.

Parameter	univariate Cox-regression		multivariate Cox-regression	
	p-value	Hazard-Ratio (95% CI)	p-value	Hazard-Ratio (95% CI)
CD3+HLADR+ frequency	<0.001	1.068 (1.037-1.099)	0.024	1.054 (1.007-1.103)
CD3+CD8+ frequency	0.107	1.022 (0.995-1.050)	0.417	0.982 (0.940-1.026)
Age	0.679	1.006 (0.979-1.033)		
Sex	0.796	0.971 (0.474-1.773)		
UICC tumor stage	0.432	1.604 (0.494-5.213)		
ECOG PS	0.012	1.977 (1.164-3.356)	0.059	1.953 (0.976-3.907)
Leukocyte count	0.521	1.020 (0.961-1.082)		
Neutrophil count	0.440	1.000 (1.000-1.000)		
Lymphocyte count	0.275	1.000 (0.999-1.000)		
NLR	0.166	1.021 (0.992-1.051)		
Hemoglobin	0.001	0.865 (0.794-0.943)	0.087	0.915 (0.826-1.013)
Sodium	0.379	0.969 (0.903-1.040)		
Potassium	0.250	0.671 (0.341-1.323)		
ALT	0.005	1.012 (1.004-1.020)	0.147	1.014 (0.995-1.033)
AST	0.023	1.012 (1.002-1.022)	0.801	0.997 (0.977-1.018)
Bilirubin	0.022	1.069 (1.010-1.131)	0.232	1.050 (0.969-1.138)
Creatinine	0.788	0.948 (0.643-1.397)		
LDH	0.777	1.000 (0.998-1.002)		

UICC, Union for International Cancer Control; ECOG PS, Eastern Cooperative Oncology Group performance status; NLR, neutrophil to lymphocyte ratio; ALT, alanin aminotransferase; AST, aspartate aminotransferase; LDH, lactate dehydrogenase.

### Frequency of CD3+HLADR+ cells during ICI treatment can predict overall survival

Consequently, we evaluated the relevance of circulating activated T cells throughout therapy using two further time points beyond the baseline: an early time-point (after only one or two cycles of therapy, n=51) and a late time-point (after three to five cycles of therapy, n=47). First, we looked at how ICI could influence the abundance of these circulating cells during therapy. By employing repeated measures ANOVA analysis comprising the three time-points we could show that there was no significant effect across all time-points (F (1.35, 48.65) = 1.686, p=0.201, **Figure 2C**), however a significant difference could be demonstrated in the *post hoc* pairwise comparison using the Bonferroni correction between the baseline and the early-time point (p=0.015). As pretreatment frequencies of activated T cells were strong predictors of OS, we next hypothesized that longitudinal values could also be prognostically relevant and serve to monitor therapy during its course. As before, we calculated optimal cut-off frequencies of circulating CD3+HLADR+ cells at the early and late-time points (frequency of CD3+HLADR+_early_: 18.0%, frequency of CD3+HLADR+_late_: 8.9%). As hypothesized, patients with a frequency of activated T-cells above the ideal cut-off survived significantly shorter than patients with frequencies below (p_early_=0.008, HR_early_: 2.790 [95%CI: 1.261-6.177], p=0.011; p_late_=0.024, HR_late_: 2.714 [95%CI:1.104-6.667], p=0.030, **Figures 2D, E**). Next, we looked at how the ICI-induced dynamics of these frequencies (increasing/decreasing between baseline and early/late time-point) predicted OS. Notably, patients with increasing frequencies of CD3+HLADR+ cells between baseline and the early as well as late time points showed a significantly improved overall survival, with patients with increasing levels living a median of 587 days (Δ baseline/early time-point) and for Δbaseline/late time-point not reaching their median OS, while patients with decreasing frequencies of activated T cells had a median OS of only 162 and 292 days, respectively (p_early/baseline_=0.019, HR_early/baseline_: 2.338 [95%CI: 1.126-4.854], p=0.023, p_late/baseline_=0.032, HR_late/baseline_: 2.423 [95%CI: 1.052-5.576], p=0.018, **Figures 2F, G**).

### Frequency of CD3+HLADR+ cells predict toxicity, response and overall survival in patients undergoing monotherapy with a single ICI agent

Bearing in mind that, in our study, patients undergoing dual blockade contribute majorly to the significant increase in CD3+HLADR+ cell frequencies after the first cycle of therapy (**Figure 2C**, **Supplementary Figure 2G–H**), we have performed an analysis of response, OS and toxicity in patients undergoing ICI monotherapy (n=64) (**Supplementary Table 2**). As seen in the table, we could show that the role of CD3+HLADR+ cell frequencies in the peripheral blood towards predicting response (p_3months_=0.089, p_6months_=0.016), and toxicity (p=0.109) and OS remains (Ideal cut off: 18.5%, median OS 587 vs. 132 days, p<0.001, HR: 5.003 [95%CI: 2.308-10.845], p<0.001), even when regarding longitudinal values and their dynamics (p_early_<0.001, HR_early_: 4.508 [95%CI:1.860-10.925], p_late_=0.031, HR_late_: 2.640 [95%CI:1.056-6.603]; p_early/baseline_=0.035, HR_early/baseline_: 2.240 [95%CI: 1.038-4.830], p=0.040, p_late/baseline_=0.038, HR_late/baseline_: 2.410 [95%CI: 1.024-5.673], p=0.044), despite the exclusion of patients undergoing dual immune checkpoint blockade (n=6). The calculated ideal cut-offs using the Charité cut-off finder are the same for both patient populations (single agent vs. all). We then compared OS using Kaplan Meier estimates of monotherapy vs dual therapy patients, showing no significant differences (p=0.677).

### An immune signature score comprising CD3+HLADR+ cell frequencies and the neutrophil-to-lymphocyte ratio is a highly significant OS predictor

**Figure 3:**
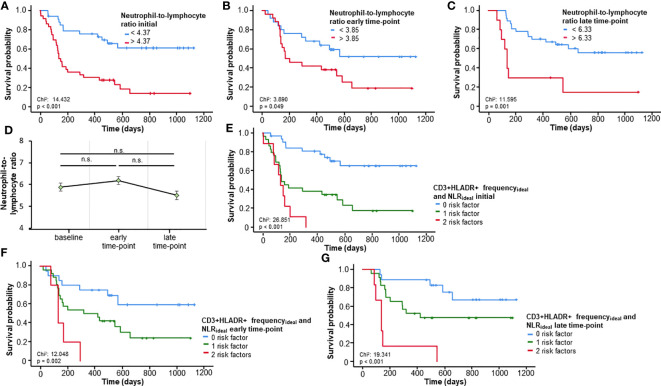
Prognostic relevance of the neutrophil-to-lymphocyte ratio (NLR) and a prognostic immune signature score including CD3+HLADR+ cell frequencies. **(A-C)** A NLR above the respective ideal cut-off value at baseline or the early/late time-point is associated with a significantly impaired overall survival (OS). **(D)** The NLR remains unaltered over time (error bars indicate SEM). **(E)** A novel immune signature score comprising baseline frequencies of CD3+HLADR+ cells and the NLR significantly predict OS. **(F, G)** The combined immune signature score shows a strong prognostic relevance for the early and late time-points. n.s.: not significant (p>0.05).

The NLR, a well investigated biomarker for patients undergoing ICI (6), was validated in our cohort as a predictor of OS for all three time-points (baseline, early and late time-points), when using its respective ideal cut-off value (NLR_baseline_:4.37, p_baseline_<0.001; NLR_early_:3.95, p_early_=0.049; NLR_late_:6.33, p_late_=0.001; **Figure 3A–C**). Repeated measures ANOVA analysis demonstrated no significant change in NLR across all three time-points (F (2, 92) = 1.053, p=0.353, **Figure 3D**), Based on these results and the findings above related to CD3+HLADR+, we established an immune signature score combining CD3+HLADR+ and NLR and how it could predict OS. In this case, frequencies of CD3+HLADR+ and NLR above the ideal cut-off were seen as risk factors. Patients bearing e.g., two risk factors had a significantly shorter OS (median OS 132 days) compared to patients with no risk factors (median OS not reached) (p<0.001, HR: 12.454 [95%CI: 4.221-36.749], p<0.001, **Figure 3E**). This score could also significantly predict OS at the early and late-time points of therapy (p_early_=0.002, HR_early_: 2.000 [95%CI: 1.120-3.571], p_early_=0.019; p_late_<0.001, HR_late_: 2.143 [95%CI: 1.157-3.970], p_late_=0.015, **Figures 3F, G**).

### Correlation between CD3+HLADR+ frequencies, T cell subsets and gut microbiome taxa

**Figure 4:**
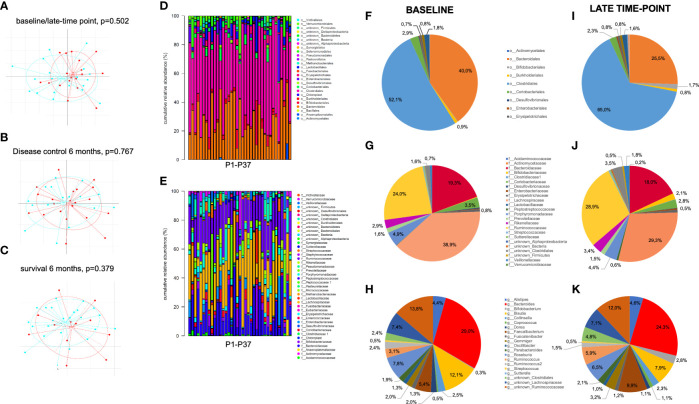
Overview of gut microbiota analyses in stool samples of patients before and after initiation of ICI treatment. **(A-C)** Beta-diversity across gut microbioma samples did not significantly differ between **(A)** pretherapeutic (blue) and late time-point (red), **(B)** responders (blue) vs. non-responders (red) and **(C)** patients who were still alive (blue) vs. deceased (red) at 6 months of ICI therapy. **(D, E)** Taxonomic binning at order **(D)** and family level **(E)** shows comparable relative abundance of different taxa across all 37 pretherapeutic samples. **(F-K)** Comparison of baseline to late time-point relative abundance of bacterial orders **(F, I)**, families **(G, J)** and the most frequent genus **(H, K)** show non-significant shifts of bacterial composition between both time points.

In a latter step, we took a glance at how the prognostically highly relevant CD3+HLADR+ cell frequencies correlated with other immune status parameters, clinical parameters and relative abundance of taxa from the gut microbiome measured by 16s rRNA sequencing. **Figure 4** depicts an overview of gut microbiome analyzes, with patients showing no significant different beta-diversity related to time-point, response and survival at 6 months therapy (**Figure 4A–C**). Furthermore, relative abundances of specific taxa do not differ significantly between baseline and late-time point, despite interesting shifts in the proportions that some taxa represent within the gut microbiome. At the order level, the proportion of Bacteroidales decreased from 40% before therapy to about 25.5% at the late time point, while Clostridiales, which before therapy represented 52.1% of all orders, increased to 65.0% at the late time-point. At a family level, Lachnospiracae represented 38.9% before therapy, a proportion which decreased to 29.3% after treatment (**Figure 4D–L**).

Regarding the immune status, frequencies of CD3+HLADR+ cells significantly correlated with the frequency of CD3+CD8+ cells (p<0.001, r_s_=0.521, **Supplementary Figure 3A**), and the frequency of CD3+CD4+ cells (p=0.002, r_s_=-0.360, **Supplementary Figure 3B**). Concerning clinical parameters, CD3+HLADR+ cell frequencies correlated with the ECOG performance status (p=0.005, r_s_=0.329, **Supplementary Figure 3C**). Finally, with regards to the measured microbiome taxa, the presence of activated T cells in the peripheral blood correlated with the relative abundance of the order Burkholderiales (p=0.006, r_s_=-0.474, **Supplementary Figure 3D**). At a deeper taxa level inside this order, these frequencies showed further correlations to the family Sutterellaceae (p=0.001, r_s_=-0.628, **Supplementary Figure 3E**) and within it the genus Sutterella (p=0.010, r_s_=-0.474 **Supplementary Figure 3F**). Furthermore, a significant correlation could be established to the Genus Bacteroides (p=0.029, r_s_=-0.365 **Supplementary Figure 3G**). More detailed values on these taxa are depicted in **Supplementary Table 1**.

### Baseline CD3+CD8+ cell frequencies also play a role in toxicity, response and overall survival prediction to ICI therapy, yet inferior to CD3+HLA-DR+ cell frequency

Since CD3+HLA-DR+ cell frequencies positively correlate significantly with CD3+CD8+ cell frequencies in our cohort, while negatively correlating with CD3+CD4+ cell frequencies, we postulate that most of these CD3+HLA-DR+ cells are indeed CD3+CD8+HLA-DR+ cells. To further investigate this aspect, we looked at the role that baseline CD3+CD8+ cells play regarding response, toxicity and OS prediction for patients in our cohort (**Supplementary Table 3**). CD3+CD8+ baseline cell frequencies seem to play a role in predicting disease control at 3 and 6 months (p=0.044 and p=0.026 respectively), toxicity of all grades (p=0.025), as is the case for CD3+HLA-DR+ cell frequencies. Also, by calculating an ideal cut-off for CD3+CD8+ cell frequency (23.65%) it is possible to discriminate between short- and long-term survivors, with patients with cell frequency values above 23.65% at baseline surviving a median of only 170 days compared to 658 days for patients below this value (HR: 2.323 [95%CI: 1.221-4.418], p=0.010). However, when using univariate Cox regression analysis, CD3+CD8+ cell frequencies at baseline do not pose as an independent predictor of overall survival, contrarily to CD3+HLA-DR+ cells (UVA: p=0.107). When adding CD3+CD8+ cells to the multivariate analysis, the independent prognostic power of CD3+HLA-DR+ cells is unaffected (HR: 1.054 [95%CI: 1.007-1.103], p=0.024, **Table 2**). Notably, despite the fact that the strong positive correlation between these two cell populations is not only present at baseline (p<0.001, rs=0.521) but also at early (p=0.004, rs=0.397) and late time-points (p=0.001, rs=0.484), contrarily to longitudinal values of CD3+HLA-DR+ and their dynamics that pose as predictors of overall survival through the course of therapy as shown above, longitudinal values of CD3+CD8+ and their dynamics don’t show any type of predictive value, even when calculating an ideal-cut off (p_early_=0.4, plate=0.056, p_early_/baseline=0.319, p_late_/baseline=0.995).

### Relative abundance of specific gut microbiome taxa associated with CD3+HLADR+ cell frequencies can significantly predict overall survival

**Figure 5:**
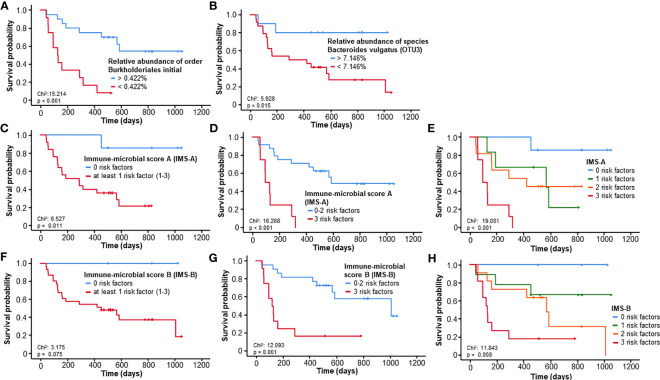
Prognostic relevance of specific gut microbiome taxa associated with CD3+HLADR+ cell frequencies. **(A, B)** A relative abundance of bacteria from the Burkholderiales order or Bacteroides vulgatus species below the ideal cut-off is associated with a significantly impaired outcome. **(C–E)** The immune-microbial score A (comprising ideal cut-offs of the NLR, the frequency of CD3+HLADR+ cells and the rel. abundance of the Burkholderiales order) is a strong predictor of survival in patients undergoing ICI therapy. **(F–H)** The immune-microbial score B (comprising ideal cut-offs of the NLR, the frequency of CD3+HLADR+ cells and the rel. abundance of Bacteroides vulgatus) is a strong predictor of survival in patients undergoing ICI therapy.

Here, we focused on four taxa that showed associations to the frequencies of CD3+HLADR+ cells: the Burkholderiales order, the Sutterellaceae family, Genus Sutterella and the Genus Bacteroides. Patients with a relative abundance of bacteria from the Burkholderiales order below the ideal cut-off of 0.422% lived significantly shorter (median OS 129 days) than patients with values above this cut-off (median OS not reached, p<0.001, HR: 6.219 [95%CI: 2.217-17.446], p=0.001, **Figure 5A**). Inside this order, a similar effect could be demonstrated for bacteria from the Sutterellaceae family related to an ideal cut-off of 0.405% (p=0.029, **Supplementary Figure 4A**) and within it the Genus Sutterella (Sutterella_ideal_:0.254%, p=0.032, **Supplementary Figure 4B**) The Bacteroides genus failed to pose as a significant predictor of OS (p=0.064, **Supplementary Figure 4C**), but within it, we identified a prognostically significant OTU representing the species Bacteroides (B.) vulgatus (OTU3). Patients with a relative abundance of B. vulgatus above the ideal cut-off (7.146%) lived significantly longer (p=0.015, HR: 5.153 [95%CI: 1.186-22.397], p=0.029, **Figure 5B**) than patients below this value. When looking at these prognostically relevant taxa in a follow-up stool sample after three to five cycles, these showed no prognostic relevance, possibly due to the low sample number (n=15). Nonetheless, patients with a relative abundance of B. vulgatus below the median (4.63%) at the late time-point showed a tendency towards better OS (p=0.092, **Supplementary Figure 4D**). In addition, also a tendency towards improved survival could be shown in patients with decreasing relative abundance of B. vulgatus between baseline and the late time-point (p=0.132, **Supplementary Figure 4E**).

### A combined immune-microbial score including activated T cells, NLR and the relative abundance of Burkholderiales order or Bacteroides vulgatus species has an important prognostic role

We then proceeded to develop two immune-microbial scores (IMS), one at the order level, involving the ideal cut-offs of the relative abundance of Burkholderiales, the NLR and frequencies of CD3+HLADR+ cells, and one at species level, involving the relative abundance of B. vulgatus, NLR and CD3+HLADR+. Concerning the first score (IMS-A), patients with at least 1 risk factor already had a significantly impaired overall survival (p=0.011, HR: 8.914 [95%CI: 1.181-67.292], p=0.034, **Figure 5C**). An even more relevant discrimination could be achieved for patients with 3 risk factors vs patients with less (p<0.001, HR: 6.732 [95%CI: 2.352-19.272], p<0.001, **Figure 5D**). **Figure 5E** shows a depiction of all 4 groups and how each risk factor contributes to a further deterioration of OS (p=0.001, HR: 2.681 [95%CI: 1.513-4.750], p=0.001). For the second score (IMS-B), patients with at least 1 risk factor did not live significantly shorter (p=0.075, **Figure 5F**). However, a significant difference could be seen in patients with all 3 risk factors, which had an impaired median OS of 120 vs. 1009 days for less than 3 or no risk factors (p=0.001, HR: 4.853 [95%CI: 1.843-12.778], p=0.001, **Figure 5G**). Again, all 4 groups can be significantly distinguished from each other (p=0.008, HR: 2.625 [95%CI: 1.440-4.785], p=0.002, **Figure 5H**).

## Discussion

To this day, extensive studies around possible biomarkers for ICI therapy have been performed, from invasive tissue-based approaches such as PD-L1 scoring and the study of the tumor microenvironment *via* profiling of co-inhibitory or co-stimulatory receptor expression *in situ* (28, 29), to minimally invasive ones studying different immunomodulators such as cytokines and cell frequencies such as the NLR in the peripheral blood (30), genetic profiles including tumor mutational burden (TMB) and microsatellite instability (MSI) (31) as well as a recently acknowledged key player in the immune system, the gut microbiome, whose manipulation through dietary interventions and FMT might impact response to ICIs (7, 16). So far, despite several candidates, only PD-L1 scoring and the TMB have found regular clinical use, still bearing some limitations (32).

In the present study, we show how easily measurable peripheral blood frequencies of CD3+HLADR+ (activated T) cells can serve as a notable predictor of response, outcome and possibly toxicity in patients undergoing immune checkpoint blockade in advanced solid malignancies. Furthermore, we show an unprecedented liaison between these cells and some microbial taxa residing in the gut of patients.

HLA-DR is an MHC-II class molecule expressed by APCs and is seen as a late activation marker for T cells, that is upregulated 48 hours after mitogen stimulation (8). Several studies have shown the role of high levels of HLA-DR+ T cells in HIV (33), autoimmune disease (34) and transplant rejection (35). In cancer, the presence of HLA-DR+ T cells in the peripheral blood has shown dichotomic results. Higher pretreatment frequencies of CD8+HLADR+ in breast cancer predicted better outcome to neoadjuvant chemotherapy (13). In squamous cell carcinoma of the lung and head and neck cancer, the same higher levels of circulating activated T lymphocytes predicted impaired overall survival (36, 37). In the ICI setting, little is known related to the biomarker role of peripheral CD3+HLADR+ cells, but Carlisle et al. report how an increase of a similar cell population after the first cycle of immunotherapy with ICI predicts better progress free-survival (PFS) and OS in RCC (14). In our cohort, we demonstrate how the peripheral blood CD3+HLADR+ frequencies can represent strong predictors of OS, with patients with higher pretreatment levels of this molecule having an impaired response and OS to ICI therapy. When frequencies of this cell population were above an ideal cut-off, patients were at a 4.5-times higher risk of impaired overall survival. We hypothesize that these high pretreatment levels support the model of a dysregulated immune system and within the CD3+HLADR+ population, some cells may have impaired antitumor immunity as suggested before (38, 39), which possibly cannot be reverted by immune checkpoint blockade. In addition, patients with a higher frequency of these activated T cells before therapy also have significantly less toxicity of any grade. Toxicity and response have been shown to go hand in hand in immune checkpoint blockade with patients with an immune system more prone to successful antitumoral directed activation by ICI demonstrating more side effects and an improved outcome (40), as is the case in our cohort. Further sustaining our thesis of dysregulated immune response and more erratic inflammation symbolized by higher pretreatment frequencies of activated T cells, ECOG status showed a strong correlation to pretreatment CD3+HLADR+ and a significant effect on OS. Not only pretreatment, but also sequentially assessed CD3+HLADR+ cell frequencies predict OS. Rather than the static CD3+HLADR+ frequencies, that can also likely depict activated T cells with impaired function unable to contribute to the antitumoral response, we also demonstrate how the dynamics of these cells, comparing early and late time-points to the baseline, can also significantly predict OS. Patients with increasing levels of CD3+HLADR+ levels as an immediate result of the first cycle(s) of immunotherapy, showing an ICI-mediated activation of T cells in the peripheral blood, which may consequently transit to the tumor microenvironment and contribute to enhanced antitumoral response, had a significantly prolonged OS, in line with prior findings (14). Interestingly, this effect was most pronounced in patients undergoing dual immune checkpoint blockade with nivolumab and ipilimumab. However, analyses excluding patients undergoing dual immune checkpoint blockade (n=6), using only patients under monotherapy (n=64), show an unaltered predictive and prognostic prediction power of CD3+HLA-DR+ cell frequencies at baseline and during ICI therapy. We also hypothesize that our CD3+HLADR+ cells are mostly CD8+ cells, since there is a significant positive correlation between both, while CD3+HLADR+ cells in our cohort negatively correlate with CD4+ cell frequencies. Our analysis concerning the predictive and prognostic prediction power of CD3+CD8+ cell frequencies shows a role for this cell population in the baseline, which cannot be verified with univariate analysis and when regarding longitudinal frequencies. Furthermore, the inclusion of this cell population in the multivariate analysis leaves the independent prognostic power of CD3+HLA-DR+ cell frequencies at baseline unaltered. Thus it is evident, that the prognostic relevance of CD3+HLA-DR+ is at least partly specific for this cell population and does not only reflect the CD8+ cell subset. Because of the complexity of the innumerous players within the immune system and the even broader individual cancer patient network (CPN), one single parameter is prone to high fluctuation between cohorts. To tackle this issue, we further analyzed a combined immune status score comprising frequencies of CD3+HLADR+ cells and a well-studied biomarker within the ICI framework, the NLR. The neutrophil to lymphocyte ratio has been shown to act as a potent prognostic predictor in ICI therapy in several cohorts, being a part of different immune signature scores shown before such as the Gustave Roussy score (41). Neutrophils have been described as facilitators of tumor growth and metastasis and stimulators of tumor angiogenesis (42). Our presented score can serve as an even better prognostic biomarker than the frequency of activated T cells by itself, with patients with values above the ideal cut-offs for both parameters before initiation of ICI treatment being at a 12.5-times higher risk of death than patients with values below the cut-offs for our cohort of patients. This immune status-based score can also serve as a biomarker to monitor therapy, also predicting OS of patients undergoing immune checkpoint blockade at an early and a late-time point during therapy.

Another important aspect of our study is the gut microbiome. We show how gut bacteria with significant correlations to the frequency of activated T cells in the peripheral blood belonging to the order Burkholderiales all the way to its genus Sutterella can successfully predict better OS in patients undergoing ICI therapy. Also, bacteria belonging to the species Bacteroides vulgatus were identified as a potential biomarker for outcome prediction in this setting, despite its genus Bacteroides only showing a trend towards better OS in patients with higher relative abundance of these bacteria. As already dissected by many reviews, the gut microbiome, despite its irrefutable role in immune modulation and its influence in cancer immunotherapy, is highly prone to fluctuation with many different studies reporting different prognostically relevant taxa, due to factors such as geography and different enterotypes, lifestyle and diet, different techniques to analyze samples and reference databases (43, 44). The relative abundance of the Burkholderiales order has been shown to impact relapse free survival (RFS) in lung tissue after resection of stage II cancer (45). In the gut, some genus inside this order have been shown in the past as successful predictors of OS, such as Burkhorderiales spp., whose supplementation lead to recovery of response to anti-CTLA4 treatment in melanoma mice by inducing interleukin 12 (IL-12)–dependent TH_1_ immune responses (46), whilst results concerning another genus within this order, Sutterella, are controversial, with one study showing how, contrarily to our findings, higher relative abundances could predict worsened OS in a NSCLC cohort undergoing ICI therapy (47), whilst favorable manipulation of the microbiome by Diosgenin therapy improved OS in patients with melanoma undergoing ICI therapy by increasing the relative abundance of the Sutterella genus (48). Little is known regarding the interaction of the Sutterella genus with the immune system, however it seems to exercise a mild pro-inflammatory activity, which we theorize could be beneficial towards immune system activation within ICI therapy, and its adhesion capacity to intestinal epithelial cells might suggest it has a immunomodulatory role (49). Some Bacteroides species play an anti-inflammatory role *via* recruitment of regulatory T-cells (Tregs), suppression of IL-17 and increase of anti-inflammatory IL-10 (50, 51). In addition, supplementation of Bacteroides spp. in melanoma mice lead to enhanced antitumoral effects and improved response to anti-CTLA-4 treatment (46). Here, we show how a higher relative abundance of the genus Bacteroides, which correlates negatively with CD3+HLADR+ cell frequencies, points non-significantly towards better OS and show how a certain species, Bacteroides vulgatus, can significantly predict OS. Interestingly, the negative correlation between Bacteroides in the gut and HLADR+ T cells in the peripheral blood can show how these possibly modulate T cell function by contributing to Treg recruitment and consequently to lesser activation of T cells. Since high levels of CD3+HLADR+ cells in a pretreatment setting seem to be unfavorable towards OS, it makes sense that high relative abundances of Bacteroides vulgatus, which can control the exaggerated presence of activated and somehow erratic T cells and the immune dysregulation some of them might represent, contribute to an improved OS in patients undergoing immune checkpoint blockade for advanced solid cancer, as shown before in a mouse model (52). Nevertheless, increasing CD3+HLADR+ cell frequencies after commencement of ICI therapy led to improved OS, since these most likely represent functional and active T cells that can combat the tumor effectively and are recruited to the TME as a result of checkpoint blockade. Simultaneously, a tendency towards better OS could be shown for patients with decreasing relative abundance of Bacteroides vulgatus after therapy initiation, most likely due to decreasing Treg recruitment, cells that result in impaired response to ICI therapy (53). In line with these findings, at a late time-point (after five cycles), a lower relative abundance of these bacteria also points towards improved outcomes. Finally, our immune-microbial scores (IMS-A and B), taking into account different factors of the complex interplay of the CPN such as CD3+HLADR+ frequencies, the NLR and microbial taxa (A: order Burkhorderiales, B: species Bacteroides vulgatus), serve as highly effective, unprecedented biomarkers in the prediction of outcome for patients with diverse advanced solid malignancies under different ICI agents.

In terms of limitations, the lack of other therapies besides ICI that our patient population is exposed to does not allow us to state whether CD3+HLADR+ cells and the presented microbial taxa as biomarkers are ICI specific or may also play a role in chemotherapy, radiotherapy or resection. In addition, the heterogeneity of our single-center patient cohort, where different cancer entities under different ICI agents are present, is one of our main limitations. Nonetheless, this same heterogeneity deems our patient population as a pan-cancer cohort, where the above depicted biomarkers show significance across a wide spectrum of malignancies and ICI drugs. Furthermore, the single-center design allows a more comprehensive and valid comparison of different demographic, clinical, radiological and laboratorial parameters across different time-points. Nevertheless, it should be noted that our analyses represent exploratory analyses only and the established cut-off values need external validation before an implementation into clinical routine could eventually be considered. Additionally, it is important to mention that since our flow cytometry data arise from a standardized and clinically established analysis by the laboratory of the hematological department of the University Medical Center Aachen, data are extracted from patient files, CD3+CD8+HLA-DR+ cell frequencies are beyond the scope of our manuscript, since their calculation does not belong to the accredited “immune status panel”. Nonetheless, we strongly believe that using the implemented standardized workflow (including the clinically validated gating strategy) from this accredited institution for these patient samples has the invaluable benefit that the generation of these results is highly comparable and any potential individual experimental bias is greatly reduced. Finally, the divergent results concerning different taxa as predictors of OS to ICI across different studies show how the gut microbiome and the enterotype are highly dynamic parameters dependent on individual characteristics such as geography and ethnicity (54), an effect that should be further explored in a multi-center design using the same sampling strategy and 16s rRNA sequencing techniques.

In conclusion, despite its needed confirmation in a larger validation cohort, this study shows the potential role of CD3+HLADR+ cells as predictors of response and toxicity in patients undergoing immune checkpoint blockade and its role in the prediction of OS in these patients before and during therapy, an effect independent of tumor entity or ICI agent. Furthermore, not only its static values but also its dynamics during ICI therapy play a significant predictive role. To better depict the complex interplay between different host immune modulators within the cancer patient network and their interaction, we present unprecedented immune-microbial scores (IMS), which can accurately predict outcome of patients with advanced solid malignancies undergoing ICI therapy. Multicenter approaches, including different therapeutic modalities (e.g. mono vs. dual immune checkpoint blockade) and larger, independent cohorts should be performed to get a better insight on the precise role of CD3+HLADR+ cells and the combined parameters.

## Data availability statement

The microbiome sequencing data presented in the study are deposited in the SRA repository, accession number is PRJNA1006689. Further raw data will be made available, upon reasonable request, and after confirming that data will be used within the scope of the originally provided informed consent.

## Ethics statement

The studies involving humans were approved by the ethics committee of the University Hospital RWTH Aachen, Germany (EK 206/09). The studies were conducted in accordance with the local legislation and institutional requirements. The participants provided their written informed consent to participate in this study. Written informed consent was obtained from the individual(s) for the publication of any potentially identifiable images or data included in this article.

## Author contributions

TL, SL, CR and JG designed the study. JG and SL recruited patients. JG and SL performed experiments. JG and SL performed statistical analysis and generated figures and tables. CB, CR, FB and TB provided intellectual input. JG, SL and TL drafted the manuscript. All authors contributed to the article and approved the submitted version.
