# Supplementary material for: Peripheral blood CD3+HLADR+ cells and associated gut microbiome species predict response and overall survival to immune checkpoint blockade
Source: Front Immunol. 2023 Aug 29;14:1206953. doi: 10.3389/fimmu.2023.1206953 (PMC10495594; doi:10.3389/fimmu.2023.1206953)

Peripheral blood CD3+HLADR+ cells and associated gut microbiome species predict response and overall survival to immune checkpoint blockade

Joao Gorgulho[1,2], Christoph Roderburg[3,4], Fabian Beier[4,5], Carsten Bokemeyer[1], Tim H. Brümmendorf[4,5], Tom Luedde[3,4,#], Sven H. Loosen[3,4,#]

[1] Department of Oncology, Hematology and Bone Marrow Transplantation with Section of Pneumology, University Medical Centre Hamburg-Eppendorf, Martinistraße 52, 20251 Hamburg, Germany.

[2] Mildred Scheel Cancer Career Center, University Cancer Center Hamburg, University Medical Center Hamburg-Eppendorf, Hamburg, Germany

[3] Department of Gastroenterology, Hepatology and Infectious Diseases, University Hospital Düsseldorf, Medical Faculty of Heinrich Heine University Düsseldorf, 40225 Düsseldorf, Germany

[4] Center for Integrated Oncology Aachen-Bonn-Cologne-Düsseldorf (CIO[ABCD]), Germany

[5] Department of Medicine IV, University Hospital RWTH Aachen, Pauwelsstrasse 30, 52074 Aachen, Germany

[#] These authors share last authorship

<u>Table of Contents</u>

<u>Supplementary Tables:</u>

- Supplementary Table 1 (page 2)

- Supplementary Table 2 (page 3)

- Supplementary Table 3 (page 4)

<u>Supplementary Figures</u>

- Supplementary Figure legends (page 5 and 6)

- Supplementary Figure 1 (page 7)

- Supplementary Figure 2 (page 8)

- Supplementary Figure 3 (page 9)

- Supplementary Figure 4 (page 10)

Supplementary Table 1: Laboratory markers, peripheral T cell subsets and gut microbiota

**Parameter**	**ICI patients** median [range]
Frequency CD3+ cells [%]	70.60 [12.50-93.30]
Absolute count CD3+ cells [cells/µl]	877.0 [185.0-2745.0]
Frequency CD3+HLADR+ cells [%]	9.10 [1.40-46.90]
Absolute count CD3+HLADR+ cells [cells/µl]	67.5 [15.0-618.0]
Frequency CD3+HLADR+ cells early time-point [%]	11.40 [2.20-40.40]
Absolute count CD3+HLADR+ cells early time-point [cells/µl]	84.0 [12.0-494.0]
Frequency CD3+HLADR+ cells late time-point [%]	10.65 [3.1-50.3]
Absolute count CD3+HLADR+ cells late time-point [cells/µl]	73.5 [12.0-519.0]
Frequency CD3+CD4+ cells [%]	40.15 [9.30-70.30]
Absolute count CD3+CD4+ cells [cells/µl]	345.0 [53.0-1090.0]
Frequency CD3+CD8+ cells [%]	22.45 [3.60-53.70]
Absolute count CD3+CD8+ cells [cells/µl]	199.5 [19.0-1375.0]
Haemoglobin [g/l]	12.20 [5.30-17.60]
Platelets [cells/nl]	245.0 [114.0-693.0]
Leucocyte count [cells/nl]	6.9 [3.1-29.1]
Neutrophil count [cells/µl]	4567.5 [1693.0-26015.0]
Lymphocyte count [cells/µl]	1216.8 [311.0-6409.0]
NLR	4.37 [0.40-40.64]
NLR early time-point	3.98 [0.31-51.17]
NLR late time-point	3.60 [1.12-39.29]
Sodium [mmol/l]	138.0 [124.0-143.0]
Potassium [mmol/l]	4.50 [3.30-6.40]
Bilirubin [mg/dl]	0.37 [0.14-3.7]
AST [U/l]	27.0 [5.0-187.0]
ALT [U/l]	19.0 [7.0-179.0]
ALP [U/l]	94.0 [35.0-1439.0]
GGT [U/l]	49.0 [9.0-1591.0]
LDH [U/l]	228.0 [8.2-1273.0]
Creatinine [mg/dl]	0.85 [0.37-3.09]
Relative abundance Order Burkholderiales [%]	0.561 [0.054-3.231]
Relative abundance Order Burkholderiales late time-point [%]	0.759 [0.001-3.728]
Relative abundance Family Sutterellaceae [%]	0.351 [0.008-3.231]
Relative abundance Genus Sutterella [%]	0.254 [0.008-3.231]
Relative abundance Genus Bacteroides [%]	13.350 [0.305-47.747]
Relative abundance Genus Bacteroides late time-point [%]	13.477 [0.009-40.696]
Relative abundance species Bacteroides vulgatus (OTU3) [%]	5.499 [0.013-24.428]
Relative abundance species Bacteroides vulgatus (OTU3) late time-point [%]	4.630 [0.001-23.166]

NLR:neutrophil-lymphocyte ratio, AST: aspartate transaminase, ALT: alanine transaminase, GGT: γ-Glutamyl transpeptidase, ALP: alkaline phosphatase, LDH: lactate dehydrogenase

Supplementary Table 2. Biomarker role of CD3+HLA-DR+ cell frequencies when taking only patients undergoing single agent ICI therapy (monotherapy) within our cohort (n=64) concerning toxicity, disease control and overall survival.

**Parameter**	**CD3+HLA-DR+ cell frequency**<br>**ICI monotherapy subcohort (n=64)**
irAE all grades	p=0.109
DC 3 months	p=0.089
DC 6 months	p=0.016
OS 3 months	p=0.072
OS 6 months	p=0.002
OS Kaplan Meier	**Baseline:**<br>Ideal cut off: 18.5%<br>Median OS 587 vs. 132 days, p<0.001,<br>HR: 5.003 [95%CI: 2.308-10.845], p<0.001<br>UVA: HR: 1.068 [95%CI: 1.037 – 1.101], p<0.001<br>MVA: HR: 1.044 [95%CI: 1.005-1.084], p=0.025<br><br>**early time-point (t1):**<br>Ideal cut off: 18.0%<br>p_{early}<0.001, HR_{early}: 4.508 [95%CI:1.860-10.925], p=0.001<br><br>**late time-point (t2):**<br>Ideal cut off: 8.9%<br>p_{late}=0.031, HR_{late}: 2.640 [95%CI:1.056-6.603], p=0.038<br><br>**Δ baseline/early time-point:**<br>Median OS 587 (increasing) vs. 162 days (decreasing)<br>$p_{early/baseline}$=0.035, $HR_{early/baseline}$: 2.240 [95%CI: 1.038-4.830], p=0.040,<br><br>**Δ baseline/late time-point:**<br>Median OS not reached vs 290 days<br>$p_{late/baseline}$=0.038, $HR_{late/baseline}$: 2.410 [95%CI: 1.024-5.673], p=0.044

irAE: immune related adverse effects, DC: disease control, OS: overall survival, UVA: univariate analysis, MVA: multivariate analysis,

Supplementary Table 3. Biomarker role of CD3+CD8+ cell frequencies concerning toxicity, disease control and overall survival.

**Parameter**	**CD3+CD8+ cell frequency**
irAE all grades	p=0.025
DC 3 months	p=0.044
DC 6 months	p=0.026
OS 3 months	p=0.066
OS 6 months	p=0.179
OS Kaplan Meier	**Baseline:**<br>Ideal cut off: 23.65%<br>Median OS 658 vs. 170 days, p=0.008,<br>HR: 2.323 [95%CI: 1.221-4.418], p=0.010<br>UVA: p=0.107; HR:1.022 [95%CI:0.995-1.050]<br><br>**early time-point (t1):**<br>Ideal cut off: 20.8%<br>p_{early}=0.4<br><br>**late time-point (t2):**<br>Ideal cut off: 20.0%<br>p_{late}=0.056<br><br>**Δ baseline/early time-point:**<br>$p_{early/baseline}$=0.319<br><br>**Δ baseline/late time-point:**<br>$P_{late/baseline}$=0.995

irAE: immune related adverse effects, DC: disease control, OS: overall survival, UVA: univariate analysis

Supplementary Figure 1.

Depiction of flow cytometry plots of whole blood preparation of 4 patients (A-D) in our cohort following staining with Panel 2 that includes the tetrachrome antibody mix CD45-FITC/CD4-PE/CD8-ECD/CD3-PC5, to which the antibody HLA-DR-PC7 was added. The gating strategy for this panel is as follows: White blood cells (WBC) are gated based on surface expression of CD45 and granularity (CD45+ and side scatter); within the white blood cell gate, lymphocytes are identified based on side scatter characteristics. From the lymphocytes gate, a quadrant gate is set based on (i) CD3 and CD4 surface staining, (ii) CD3 and CD8 surface staining and (iii) CD3 and HLA-DR surface staining. The resulting numbers in the immune status (% of CD3+CD4+ cells of all lymphocytes, %CD3+CD8+ cells of all lymphocytes and %CD3+, HLA-DR+ cells of all lymphocytes) are derived from quadrant gate A2, B2 and C2 as highlighted with the red square. % of CD3+ cells from lymphocytes are gated on the histogram as depicted. Further stainings within panel 1 (stained with the antibody mix CD45-FITC/CD56-PE/CD19-ECD/CD3-PC5, to which the antibody CD-16 PE was added) were performed to denote expression of B cells (CD19+), NK cells (CD3-CD56+CD16+) and NK like T cells (CD3+CD56+CD16+), and are not part of this gating strategy, as Panel 1 is stained separately.

Supplementary Figure 2.

Pretreatment freq. of CD3+HLA-DR+ cells do not significantly differ between different tumor entities (A), sex (B), UICC stage (C), smoking status (D), whether patients and been exposed to previous systemic cancer therapy (E). Pretreatment freq. of activated T-cells are significantly different according to ECOG PS status (F). Concerning chosen ICI drug, CD3+HLADR+ frequencies are not significantly different between patients at a pretreatment time-point when comparing dual to single immune checkpoint blockade (G) but when comparing early time-point frequencies, a significant difference can be observed with patients under Nivolumab/Ipilimumab therapy having higher levels than patients submitted to monotherapy (H).

Supplementary Figure 3.

Freq. of CD3+HLA-DR+ cells at baseline positively correlate with frequency of CD3+CD8+ (CTLs) cells (A) and ECOG status (C) and negatively correlate with the frequency of CD3+CD4+ cells (B), as well as different gut microbiome taxa (D-G).

Supplementary Figure 4.

(A-B) Patients with a relative abundance of bacteria from the family Sutterellaceae and Genus Sutterella above an ideal cut-off have significantly improved OS compared to patients below this value, whereas concerning the Genus Bacteroides only a non-significant trend in the same direction can be depicted (C). Contrastingly, patients with a late-time point relative abundance of Bacteroides vulgatus above an ideal cut-off show a tendency towards impaired OS. (D) Patients with decreasing relative abundance of B. vulgatus during therapy show a tendency towards better OS.

Supp. Figure 1

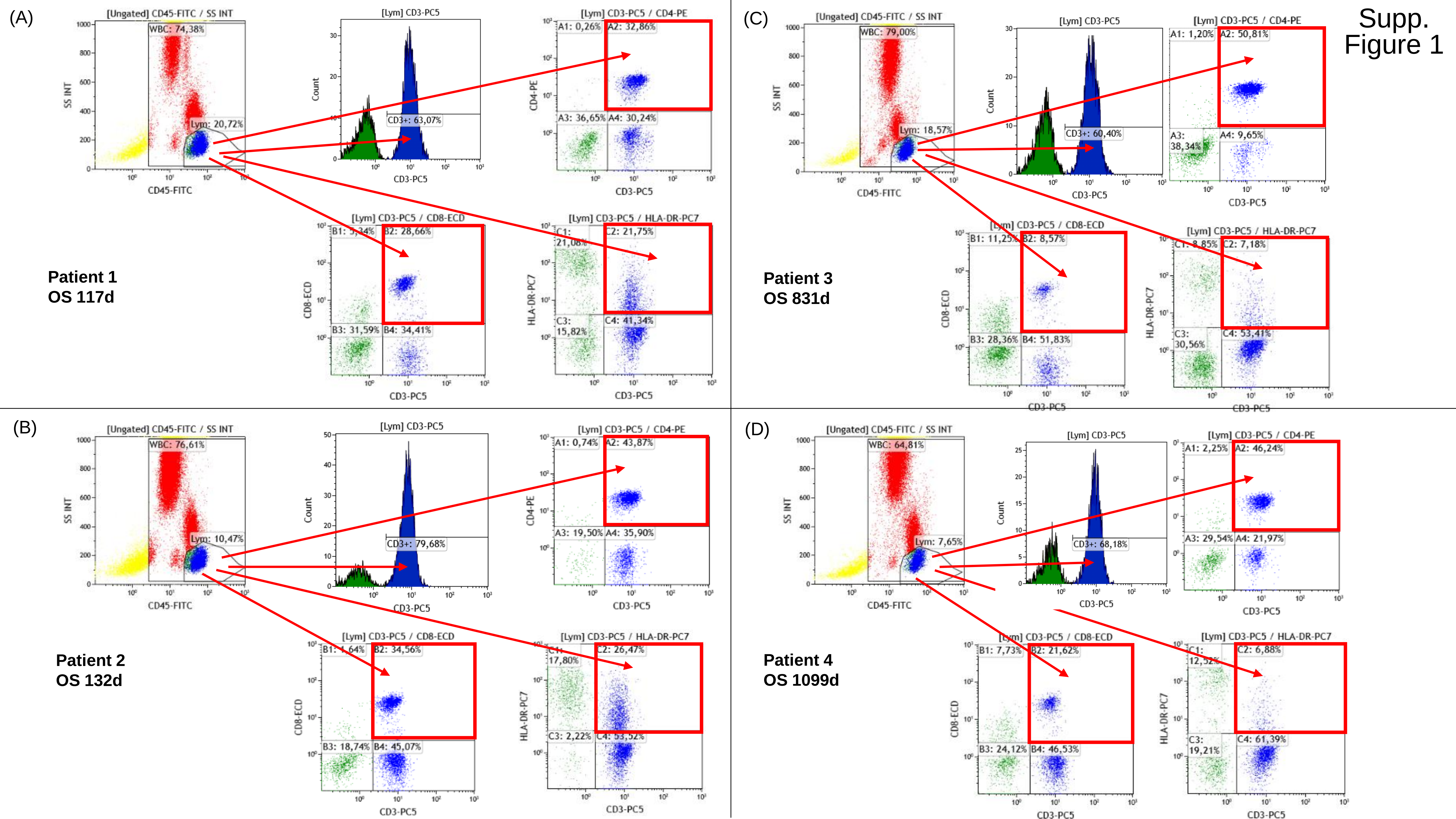

Supp. Figure 2

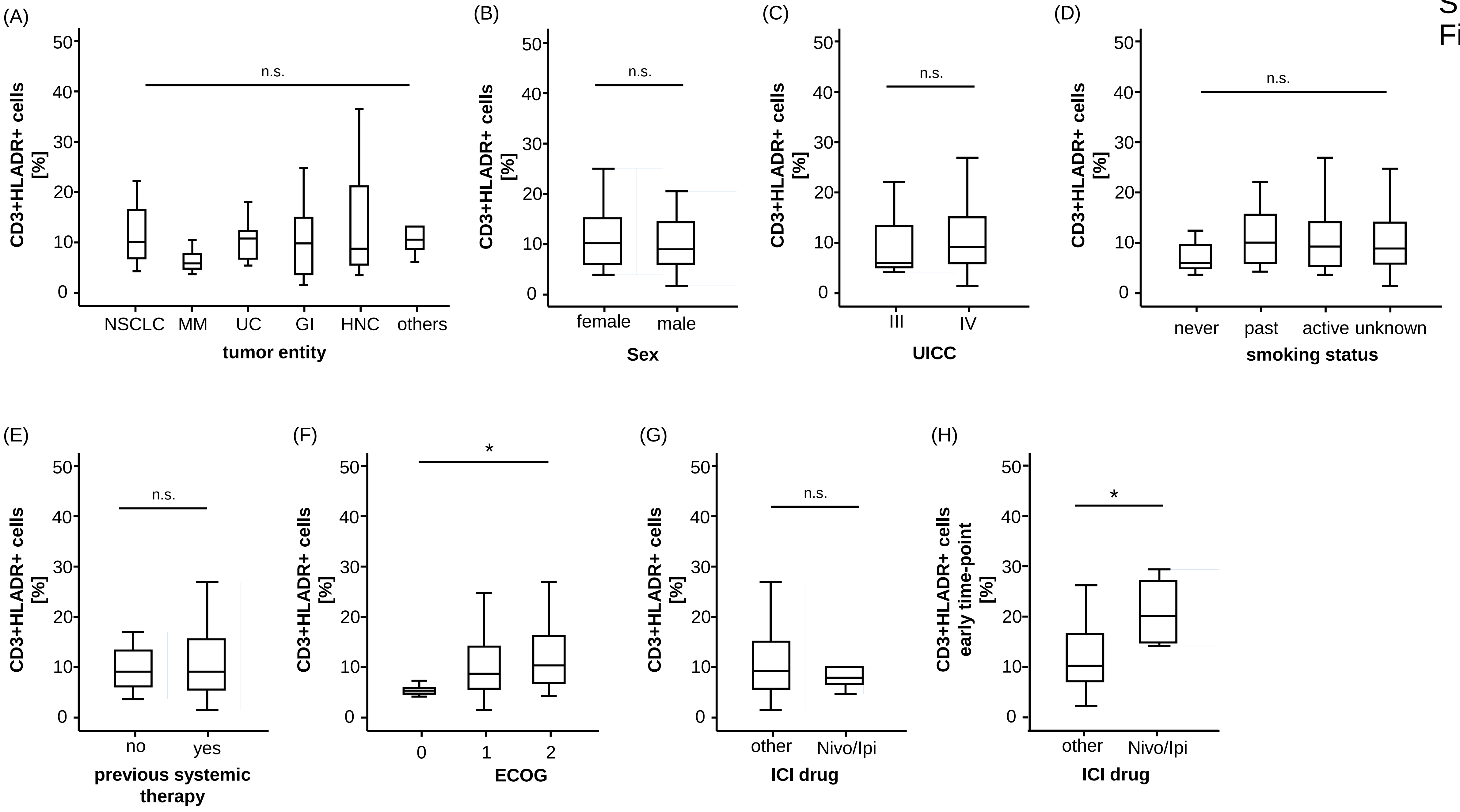

Supp. Figure 3

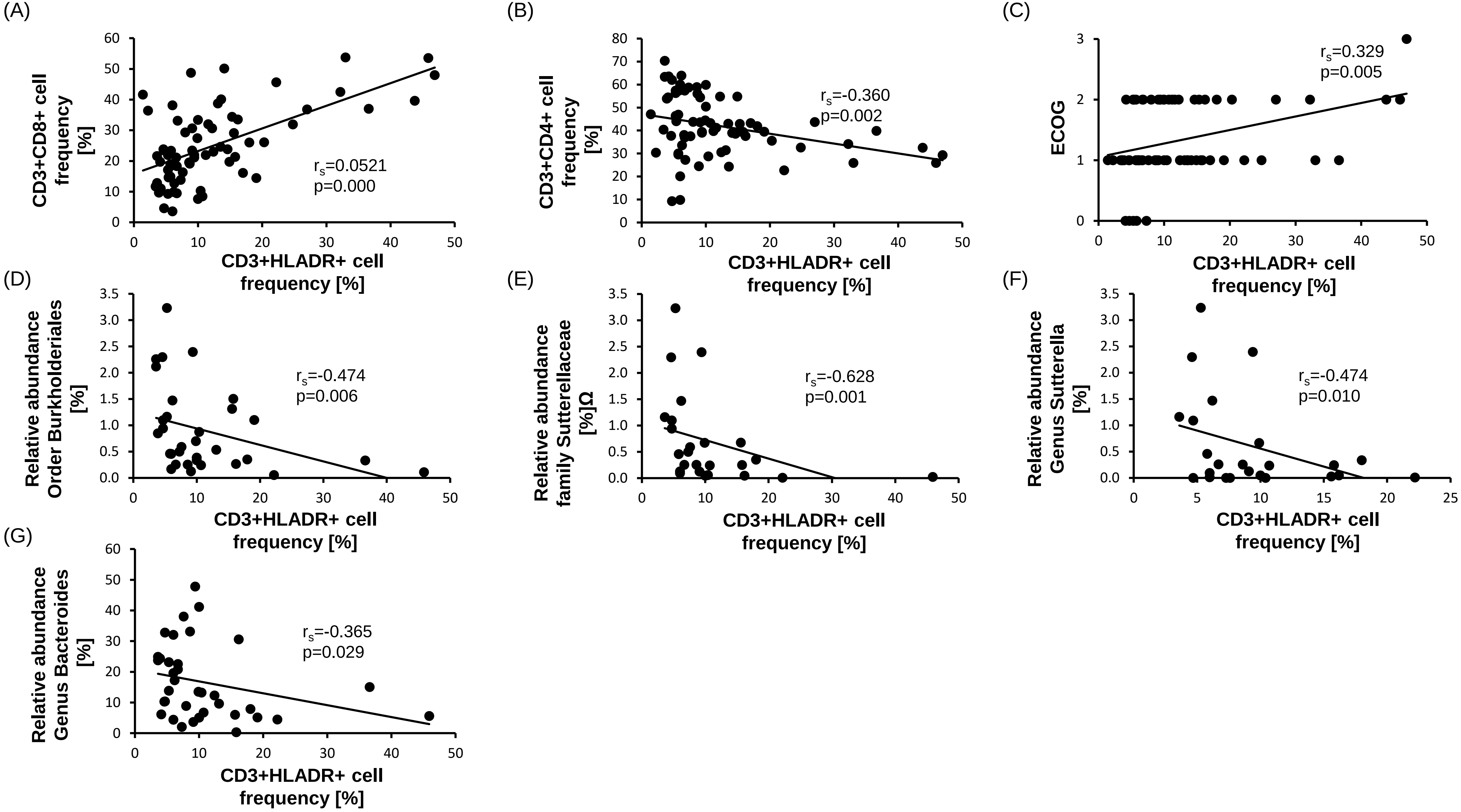

Supp. Figure 4

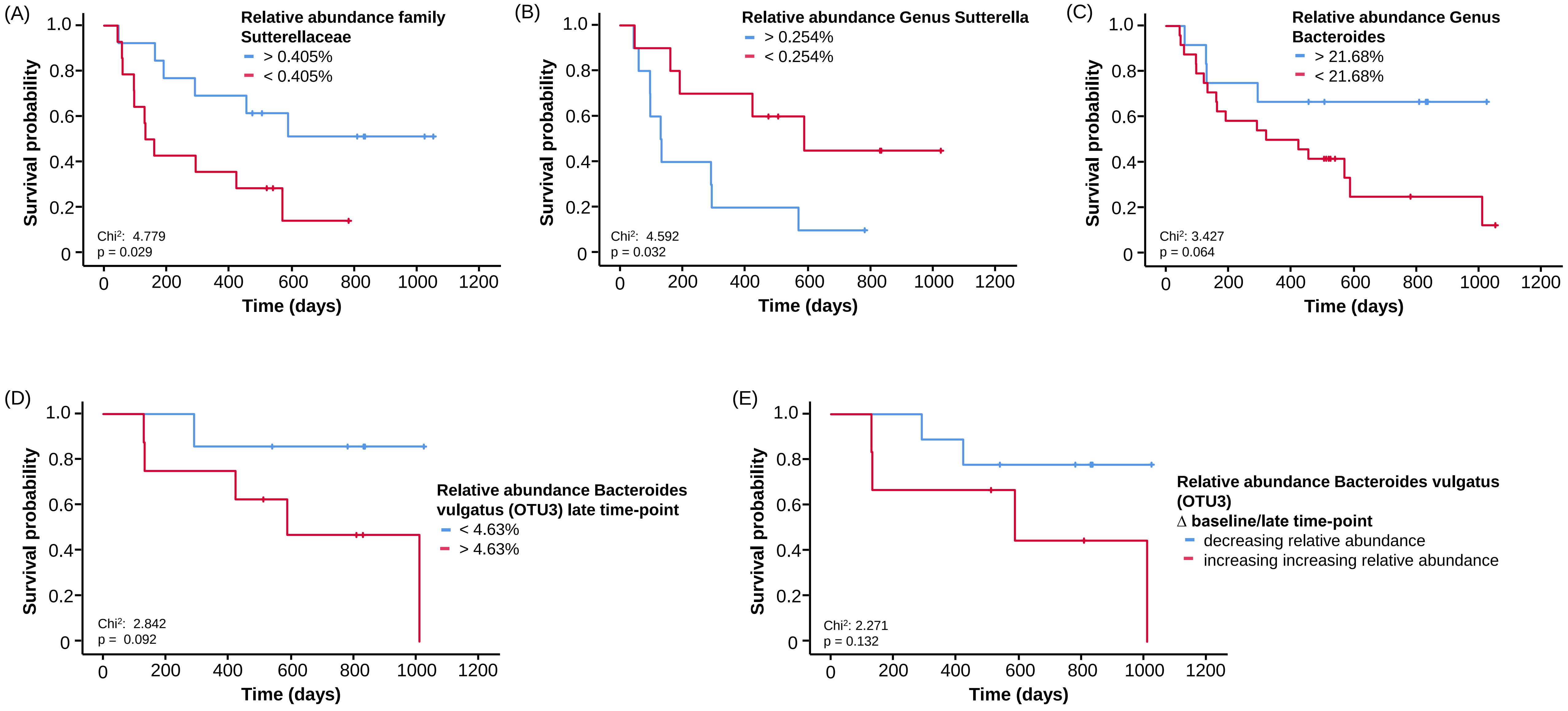

Supplement: Supplementary file 1 [file DataSheet_1.pdf]
